# Supplementary material for: STIM1‐mediated calcium influx controls antifungal immunity and the metabolic function of non‐pathogenic Th17 cells
Source: EMBO Mol Med. 2020 Jul 1;12(8):e11592. doi: 10.15252/emmm.201911592 (PMC7411566; doi:10.15252/emmm.201911592)
Supplement: Supplementary file 1 — Appendix [file EMMM-12-e11592-s001.pdf]

APPENDIX

Table of contents

1. Case reports
2. Legends to Appendix Figures S1-5
3. Appendix Tables S1-2
4. Appendix Figures S1-5
5. Appendix Table S3

1. CASE REPORTS

Patient 1 (*STIM1* p.L374P). P1 (A-II-1) was born to parents who are second cousins. He first came to medical attention in the first few weeks of life. His clinical phenotype was notable for anhidrotic ectodermal dysplasia characterized by sparse hair, the inability to sweat, intolerance of warm temperatures and dystropic nails with no evidence of fungal infection. Consistent with ectodermal dysplasia, the teeth were fragile and crumbling on eruption prompting the diagnosis of amelogenesis imperfecta. Eye abnormalities noted early after birth included non-reactive pupils and the absence of sphincter muscles in the iris with a unifying diagnosis of partial iris hypoplasia. Features of severe myopathy were noted early in life with a positive Gower's sign and muscle weakness that was worse proximally. Sensation and reflexes as well as nerve conduction tests were normal. A muscle biopsy revealed a paucity of type 2 fibres. Since his early 20's, P1 had significant cachexia with a BMI of 13.1. He required a cane to ambulate, and was unable to sit up from the examination bench unassisted.

Clinical symptoms of immunodeficiency began in early life with recurrent sinopulmonary infections as well as severe primary varicella infection with features of encephalitis and the development of new pox lesions for 4 weeks after infection onset. P1 suffered from recurrent pneumonias culminating in the development of severe bronchiectasis with clubbing. Organisms implicated in his chronic lung infections included *Mycobacterium heckeshornense, Pseudomonas aeruginosa, and methicillin resistant Staphylococcus aureus.* Laboratory testing supported the diagnosis of combined immunodeficiency (CID) with normal numbers of circulating lymphocytes and T, B and NK cells for age **(Supplemental Table 2**). Serum immunoglobulins were low-normal (although the patient did go on to receive immunoglobulin replacement, which reduced the number of infections) and he was able to generate detectable antibody titers against both tetanus and diphtheria vaccine and blood group antigens. Lymphocyte proliferation tests showed a normal response to phytohemagglutintin (PHA), pokeweed mitogen and *S. aureus* Cowan Protein A, whereas responses to varicella zoster virus, Herpes simplex virus (HSV), rubella, mumps, measles, CMV, and *C.* antigens were absent. At 23 y/o, P1 developed EBV-associated lymphoproliferative disease most consistent with a large B cell lymphoma, which presented as a masseter muscle mass. He was started on rituximab single

agent chemotherapy. Following 4 doses of single agent chemotherapy with rituximab, clinical remission was achieved for 9 months, after which his EBV viral load increased, as did his lymphadenopathy. He was treated with another 4 rounds of rituximab, with no clinical improvement. P1 passed away at 25 y/o while enrolled in paliative care from respiratory failure due to acute pneumonia, secondary to chronic lung disease and myopathy.

Patient 2 (*STIM1* p.L374P). P2 (A-II-2) is the sister of P1. Two weeks after birth, she was diagnosed with mydriasis as a consequence of a bilateral partial aniridia cumulating in severe photophobia. She further exhibited delayed motor development and poor head control with hypotonia in her first years of life. At 6 y/o, P2 suffered from a proximal muscle weakness as part of a non-specific myopathy, which was confirmed by decreased motor units with polyphasic low amplitudes and short durations in EMG measurements. A muscle biopsy from the left biceps muscle at 6 y/o revealed a predominance of type I muscle fibers and a type II muscle fiber atrophy. In addition to hypotonia, P2 further showed signs of anhidrotic ectodermal dysplasia (EDA) with sparse and thin hair, dry skin, profound anhidrosis, and enamel hypoplasia. In addition, P2 presented with arachnodactyly with hyperextensibility and swollen DIPs of the right hand during later stages of the disease (~ 7 y/o).

Signs of immunodeficiency were apparent within the first years of P2's life when she started to develop recurrent typical pneumonias, maxillary and left ethmoidal sinusitis, purulent rhinitis and a left otitis media leading to a mild-to-moderate middle ear deafness during the later disease course. Physical examinations revealed seborrheic dermatitis and recurrent monilial rashes (thrush) caused by *Candida* species. Ultrasound revealed enlarged cervical lymph nodes and hepatosplenomegaly. A liver biopsy taken at 6 y/o appeared unsuspicious. Laboratory analyses showed a microcytic hypochromic anemia. Immunoglobulin levels for IgM, IgG, IgA and IgE at 7 y/o were normal. At 28 y/o, IgM (247 mg/dL), IgA (195 mg/dL) and IgG (762 mg/dL) remained in the normal range **(Supplemental Table 2**). However, antibody titers following rubella vaccination never increased sufficiently. A chest X-ray revealed hyperinflated lungs in line with broncho-obstruction during childhood asthma. P2 was started on a treatment with inhaled steroids that evoked *Candida* skin infections including severe onychomycosis. However, lung function testing during the later disease course using a rapid acting beta-2 agonist disproved the suspected diagnosis of allergic asthma. Sarcoidosis was excluded by negative testing for angiotensin converting enzyme (ACE) and X-rays. At 26 y/o, P2 was diagnosed with Crohn's disease and treated with Remicade infusions. Upon TNF-α blockade P2 exhibited onychomycosis and recurrent left-sided urolithiasis complicated by urosepsis induced by *Clostridium difficile*. At 6 y/o, P2 presented with a failure to thrive, malabsorption, hypercholesterinemia, hypertriglyceridemia, and steatorrhea. Chymotrypsin testing and negative α-Gliadin-IgA-antibodies excluded endocrine pancreas dysfunction and coeliac disease. While esophagogastroduodenoscopy showed no abnormalities, secretin-pancreozymin stimulation revealed an exocrine pancreas dysfunction. Laboratory analyses revealed decreased

HDL and lipoprotein A levels, whereas lipoprotein B were elevated. Parathyroid hormone (PTH) and blood Ca^{2+} levels were increased and were accompanied by hypercalciuria and nephrolithiasis. Multiple Endocrine Neoplasia (MEN) syndrome was excluded based on normal thyroid-stimulating hormone (TSH) blood levels and Addison's disease was disproved by an unsuspicious adrenocorticotropic hormone (ACTH) stimulation test. At 28 y/o, P2 gave birth to a healthy boy at 36 weeks of gestation. Delivery was via Cesarean section due to HPV infection and perianal disease, both related to Crohn's disease and Remicade therapy. P2 breastfed normally until at least 3 months of age.

2. APPENDIX FIGURE LEGENDS

Appendix Figure S1. Abolished SOCE in T cells of patients with STIM1 p.L374P mutation. (A) STIM1 protein expression in fibroblasts of a healthy donor (HD) and a patient homozygous for a STIM1 p.E128RfsX9 null mutation reported previously(*34*). Representative histograms show staining of fixed and permeabilized fibroblasts with an polyclonal antibody against the C terminus of STIM1. Bar graphs show the mean fluorescence intensity (MFI) of STIM1 subtracted by the MFI of isotype IgG staining. The resulting delta MFI (MFI_{STIM1} - MFI_{IgG}) of HD cells was set to 1. Data are the mean ± SEM from 2 independent experiments. *** p<0.001. **(B)** Human T cells of P1 (red), P2 (blue), their mother (grey) and HD (black) were cultured *in vitro* for 6 weeks and analyzed for Ca^{2+} levels after stimulation with 1 μM thapsigargin (TG) (at 120s) in Ca^{2+} free Ringer's solution to deplete intracellular Ca^{2+} stores followed by addition of 1 mM Ca^{2+} to induce SOCE (at 400s). Traces represent the mean ± SEM from two measurements.

Appendix Figure S2. No proliferation defect of STIM1 p.L374P T cells cultured *in vitro*. (A,B) Proliferation of $CD4^+$ **(A)** and $CD8^+$ **(B)** T cells of P1, P2, their mother and a HD control that were stimulated *in vitro* for 6 weeks with PHA (1 µg/ml) in the presence of irradiated buffy coat cells (PBMC : feeder cell ratio of 1:1), EBV-transformed B cells (PBMC : B cell ratio of 1:10) and IL-2 (50 U/ml), loaded with CFSE and restimulated with anti-CD3 and anti-CD28 in the presence or absence of 1 μM FK506 for 1, 2 and 3 days *in vitro*. Histograms show the dilution of CFSE in T cells of P1 (red), P2 (blue), their mother (grey) and a HD (black) on day 0 (filled gray histograms) and day 1, 2, 3 of stimulation. One representative experiment of two is shown.

Appendix Figure S3. Impaired cytokine production by T cells of STIM1 p.L374P patients.
(A) Frequencies of $CD3^+$, $CD4^+$ and $CD8^+$ T cells among T cell of P1, P2, their mother and a HD control that were stimulated *in vitro* for 6 weeks with PHA (1 µg/ml) in the presence of irradiated buffy coat cells (PBMC : feeder cell ratio of 1:1), EBV-transformed B cells (PBMC : B cell ratio of

1:10) and IL-2 (50 U/ml). **(B)** Frequencies of IL-2, IFN-γ and IL-17A expressing $CD4^+$ and $CD8^+$ T cells of patients and controls after restimulation with PMA and ionomycin for 6 hours and analyzed by flow cytometry. **(C,D)** Human T cells from two healthy donors, P1 and his mother were cultured in the continuous presence of *C. albicans* and IL-2 for 2-4 weeks. T cells were restimulated with PMA and ionomycin for 6 h and analyzed for IL-2, IFNγ and TNFα production. Bar graphs represent the mean ± SEM from two independent experiments. Statistical analysis by one-way ANOVA. ***p<0.001.

Appendix Figure S4. Comparable neutrophil frequencies in the bone marrow of WT and *$Stim1^{fl/fl}$ Cd4Cre* mice. **(A)** Detection of $CD11b^+$ and Ly-$6G^+$ neutrophils in the bone marrow of WT and *$Stim1^{fl/fl}$ Cd4Cre* mice. **(B)** Bar graphs show the frequencies of $Cd11b^{hi}$ Ly-$6G^{hi}$, $Cd11b^{int}$Ly-$6G^{lo}$ and $Cd11b^{int}$Ly-$6G^{hi}$ neutrophil subsets from 4-6 mice per genotype and 2 repeat experiments.

Appendix Figure S5. Normal glycolysis, OXPHOS and mTOR signaling in STIM1-deficient T cells cultured *in vitro*. (A,B) Analysis of the extracellular acidification rate (ECAR, in mpH/min) of human T cells that were stimulated *in vitro* for 6 weeks with PHA (1 µg/ml) in the presence of irradiated buffy coat cells (PBMC : feeder cell ratio of 1:1), EBV-transformed B cells (PBMC : B cell ratio of 1:10) and IL-2 (50 U/ml) from P1 (red), P2 (blue), their mother (grey) and a HD (black). Cells were sequentially treated with 25 mM glucose, 5 µM oligomycin and 100 mM 2-DG. **(A)** ECAR traces summarized from 3 independent experiments are shown. **(B)** Bar graphs represent data from A analyzed for glycolysis (after glucose addition), glycolytic capacity, glycolytic reserve and non-glycolytic acidification ± SEM of 3 repeat experiments. **(C,D)** Analysis of oxygen consumption rate (OCR, in pmol/min) of expanded T cells from patients and controls as in A. Cells were sequentially treated with 1 µM oligomycin, 1.5 µM FCCP and 100 nM rotenone / 1 µM antimycin. **(C)** OCR traces summarized from 3 independent experiments are shown. **(D)** Bar graphs represent data from C analyzed for basal and maximal respiration, spare respiratory capacity (SRC), coupling efficiency and ATP production. Traces and bar graphs in A-D represent the mean ± SEM of 3 repeat experiments. **(E,F)** Analysis of mTOR and p70S6 phosphorylation in $CD4^+$ and $CD8^+$ T cells of P1 (red), P2 (blue), their mother (grey) and a HD (black) that were stimulated *in vitro* for 6 weeks with PHA (1 µg/ml) in the presence of irradiated buffy coat cells (PBMC : feeder cell ratio of 1:1), EBV-transformed B cells (PBMC : B cell ratio of 1:10) and IL-2 (50 U/ml), restimulated with anti-CD3/CD28 for 24 hours, left unstimulated or stimulated. Histograms represent intracellular staining of cells with anti-phospho-mTOR (Ser2448) **(E)** and anti-phospho-p70S6 (Ser235/236) **(F)** antibodies. Filled gray histograms represent unstimulated cells. Gates indicate the frequencies of $CD4^+$ and $CD8^+$ T cells positive for phospho-mTOR and phospho-p70S6 staining.

**Patient**	**P1** (A-II-1)		**P2** (A-II-2)		
***STIM1* mutation**	p.L374P		p.L374P		
**Age**	11 y/o	22 y/o	7 y/o	10.5 y/o	28 y/o
**Immune cell populations** (in x10^9/μl)					
Granulocytes	**15.61** [1.5-5.6]	**6.98** [2-6]	WBC normal	4.08 [1.5-5.6]	
Monocytes	**1.72** [< 0.5]	**1.00** [0.1-0.8]		0.47 [<0.5]	
Eosinophils	0.9 [< 0.8]	0.19 [0-0.45]		0.13 [<0.8]	
Basophils	0.04 [< 0.2]	0.06 [0-0.1]		0.05 [<0.2]	
Lymphocytes	1.81 [0.9-3.5]	1.27 [1-4]		1.95 [1.4-4.1]	
T cells			normal		
$CD3^+$	1.38 [0.80-3.50]	0.91 [0.7-2.1]			
$CD4^+$	1.03 [0.40-2.10]	0.58 [0.41-1.33]			
$CD8^+$	0.30 [0.20-1.20]	0.29 [0.2-0.78]			
B cells ($CD19^+$)	0.37 [0.2-0.6]	0.22 [0.1-0.5]	0.84 [0.18-1.52]		
NK cells ($CD3^- CD16^+ CD56^+$)	0.04	0.09 [0.09-0.6]			
NK T cells ($CD3^+ CD16^+ CD56^+$)		0.03			
**Lymphocyte proliferation** cpm (s.i.)			normal		
PHA	**5,514 (65)**	**2,686**			
Pokeweed Mitogen	17,044 (201)	2,729			
S. aureus Cowan, Protein A	1,218 (12)	568			
Candida albicans	**31 (0)**	**489 (2)**			
Cytomegalovirus	**28 (0)**	**591 (3)**			
Herpes simplex	**91 (1)**	1,812 (8)			
Measles	**344 (2)**	**702 (3)**			
Mumps	**187 (1)**	**584 (3)**			
Rubella	**60 (0)**	**422 (2)**			
Varicella Zoster	4,872 (28)	847 (4)			
**Serum Ig**					
IgM (g/L)	0.54 [0.35-2.39]		normal		247 mg/dL (40-230mg/dL)
IgG (g/L)	**5.80** [7.59-15.5]		normal		762 mg/dL (700-1600mg/dL)
IgA (g/L)	3.63 [0.58-3.59]		normal		195 mg/dL (70-400mg/dL)
IgE (μg/L)	1 [32]		normal		normal
Diphteria antibody	0.03 U/mL (low)				
Tetanus antibody	0.06 U/mL (low)				
Rubella antibody				negative	

Appendix Table S1: Immune cell populations and functions of P1 and P2. cpm, counts per minute; Ig, Immunoglobulin. Results for P1 (11 y/o) reported as cpm and stimulation index (s.i.) which represents the ratio of cpm of the stimulated lymphocytes to the mean cpm of an unstimulated control. Reference values in [ ].

Anti-human antibodies			
**Antigen**	**Manufacturer**	**Clone**	**Conjugation**
CD3	Biolegend	SK7	APC-Cy7
CD3	Biolegend	UCHT1	Alexa700
CD4	Biolegend	OKT4	BV510
CD4	Biolegend	RPA-T4	PE, PE-Cy7
CD4	BioLegend	OKT4	Alexa700
CD8	Biolegend	HIT8a	Alexa700
CD8	Biolegend	RPA-T8	PE-Cy7
CD8	Biolegend	SK1	Pacific-Blue
CD45RO	BD Pharmingen	UCHL1	PE-Cy7
CD45RO	Biolegend	UCHL1	APC-Cy7
CD44	Biolegend	IM7	PerCP
CD57	Biolegend	HCD57	Pacific-Blue
CD16	Biolegend	3G8	Alexa488
CD56	Biolegend	HCD56	PerCP-Cy5.5
CD27	Biolegend	O323	PE-Cy7
CCR7	R&D	150503	FITC
HLA-DR	Biolegend	L243	APC-Cy7
Va24Ja18 TCR	Biolegend	6B11	APC
Foxp3	BioLegend	259D	PE
IL-2	Biolegend	MQ1-17H12	Alexa700, APC
IL-22	eBioscience	22URTI	eF710
TNF-α	Biolegend	MAb11	A488
IL-17A	eBioscience	eBio64CAP17	PE
IFN-γ	eBioscience	4S.B3	Alexa488
γδ TCR	BD	B1	biotinylated
+streptavidin	Biolegend	-	BV605
ViabDye	eBioscience	-	eF506
Phopsho mTor Ser2448	eBioscience	MRRBY	eFluor660
Phospho S6 Ser235/236	eBioscience	cupk43k	APC
**Anti-mouse antibodies**			
**Antigen**	**Manufacturer**	**Clone**	**Conjugation**
CD4	eBioscience	GK1.5	PE-Cy7, eFluor450
CD44	eBioscience	IM7	Pacific-Blue, FITC
IL-2	eBioscience	JES6-5H4	FITC
IFN-γ	eBioscience	XMG1.2	APC, PE
TNF-α	eBioscience	MP6-XT22	APC
IL-17A	eBioscience	eBio17B7	APC
IL-17A	Biolegend	TC11-18H10.1	FITC
IL-17F	eBioscience	eBio18F10	PerCP eFluor710
GM-CSF	Biolegend	MP1-22E9	PerCP-Cy5.5
CD45	Biolegend	30-F11	PE-Cy7
MHC-II	eBioscience	M5/114.15.2	eFluor450
Cd11b	eBioscience	M1/70	APC
Gr-1 (Ly6G)	eBioscience	RB6-8C5	PE
Gr-1 (Ly6G)	Biolegend	1A8	AlexaFluor647

Appendix Table S2: Commercial antibodies used for flow cytometry.

Appendix Figure S1

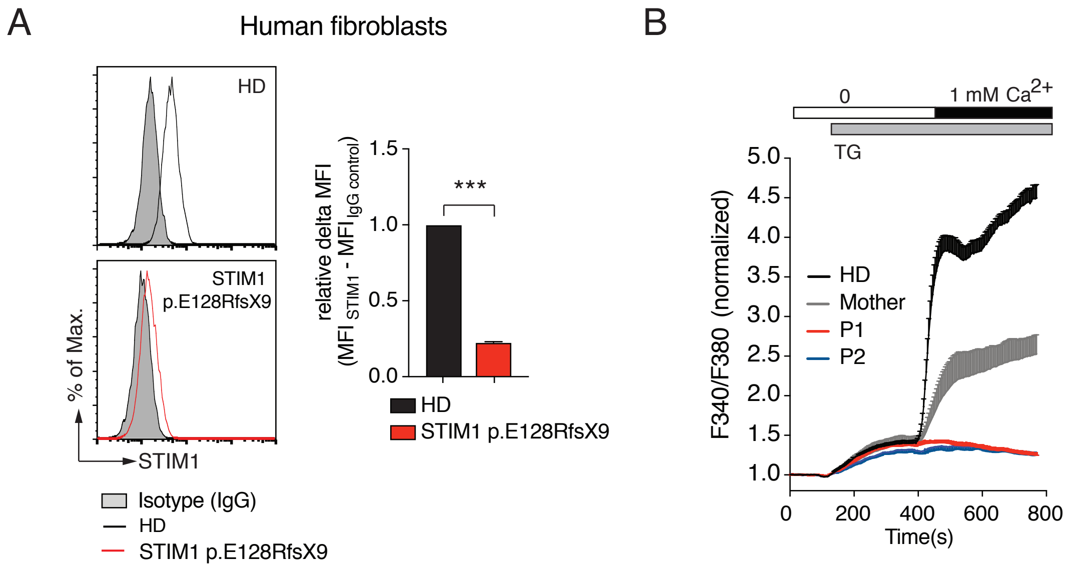

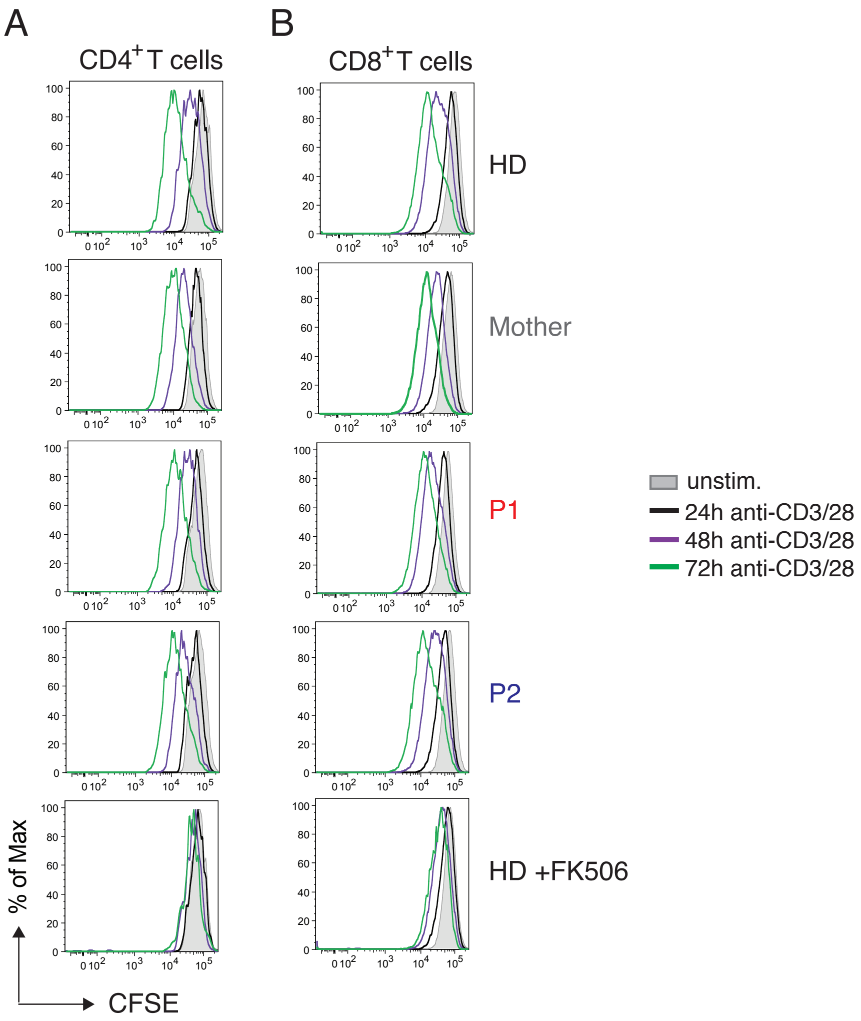
A
B
CD4+ T cells
CD8+ T cells
HD
Mother
P1
P2
HD +FK506
unstim.
24h anti-CD3/28
48h anti-CD3/28
72h anti-CD3/28
% of Max
CFSE

Appendix Figure S3

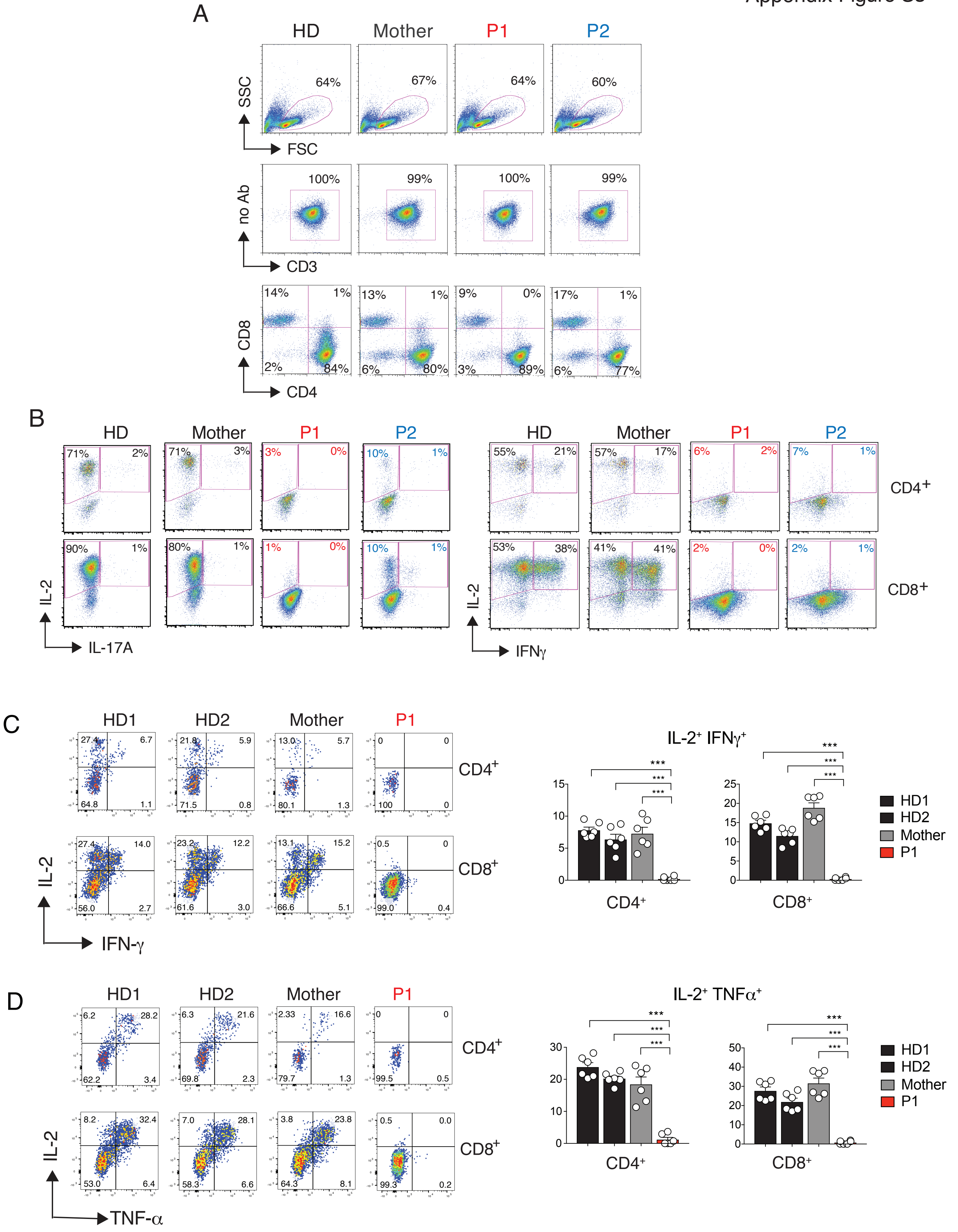

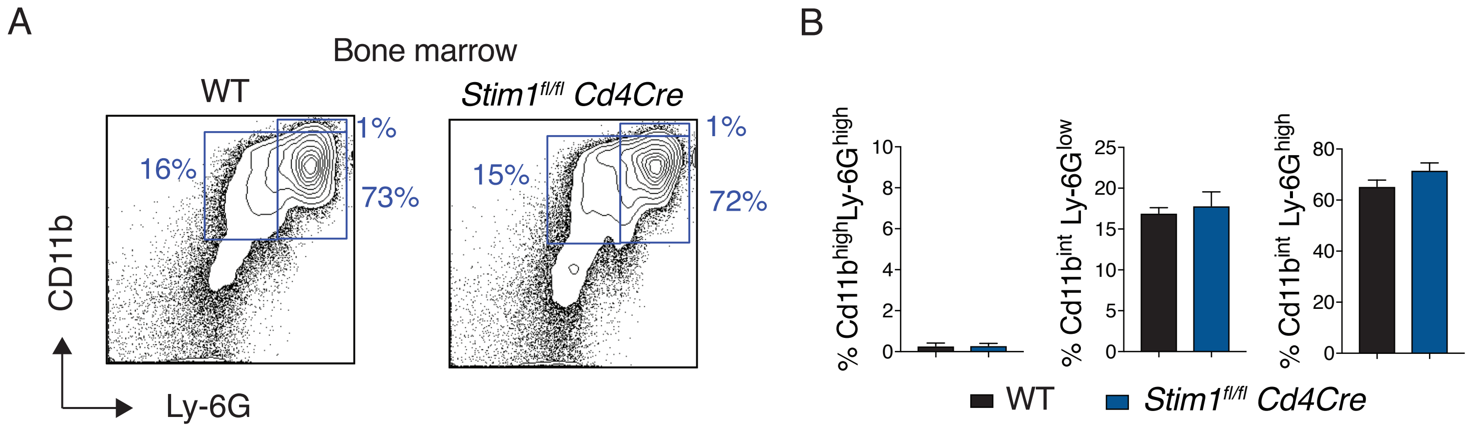
A
Bone marrow
WT
Stim1fl/fl Cd4Cre
16%
1%
73%
15%
1%
72%
CD11b
Ly-6G
B
% Cd11bhighLy-6Ghigh
% Cd11bint Ly-6Glow
% Cd11bint Ly-6Ghigh
WT
Stim1fl/fl Cd4Cre

Appendix Figure S5

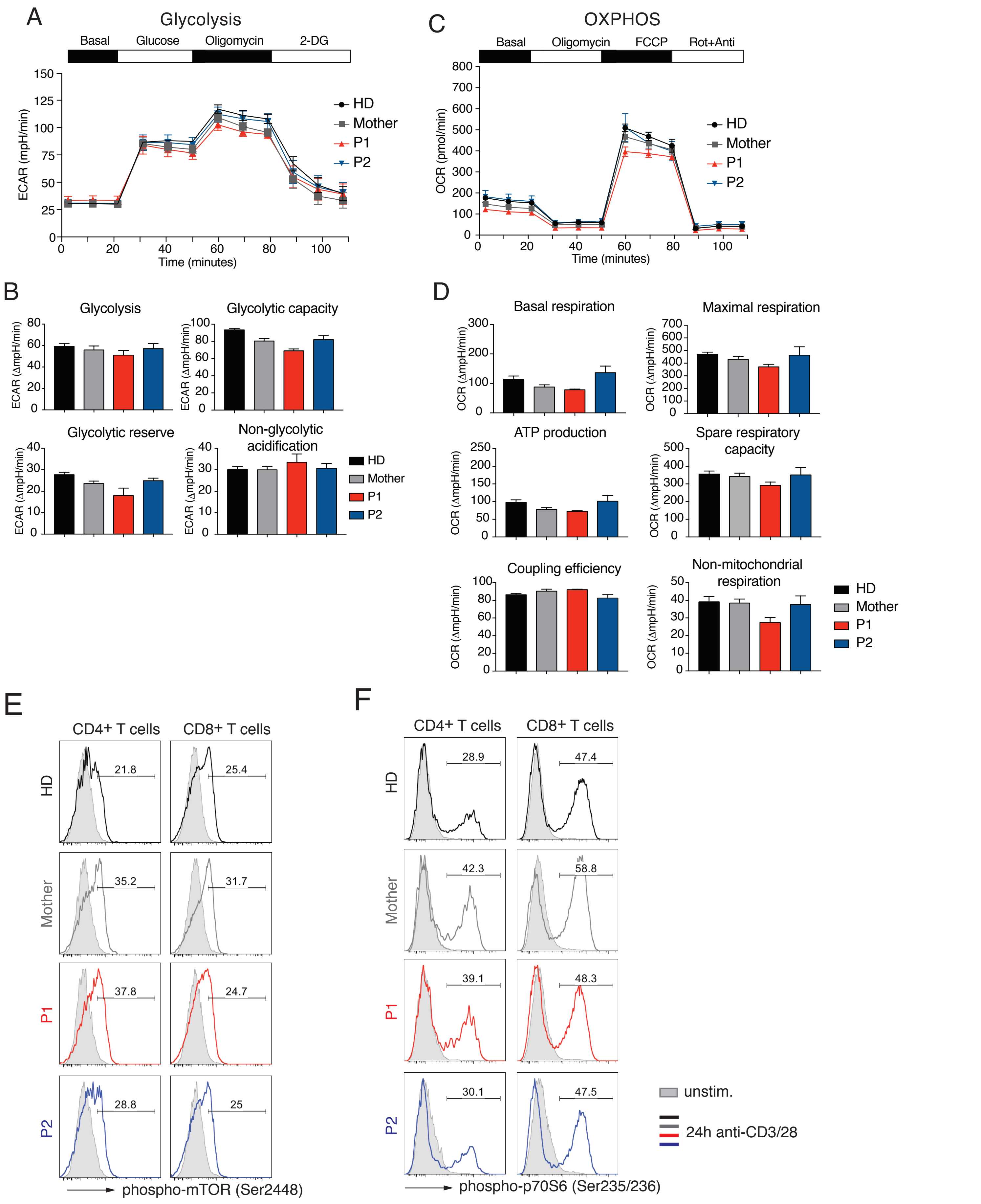

Figure panel	Statistical test	Comparison	p value
Figure 1F (AUC)	Unpaired Student’s t-test	HD versus P1	p<0.0001
	Unpaired Student’s t-test	HD versus P2	p<0.0001
	Unpaired Student’s t-test	Mo versus P1	p<0.0001
	Unpaired Student’s t-test	Mo versus P2	p<0.0001
Figure 1F (ΔPeak)	Unpaired Student’s t-test	HD versus P1	p<0.0001
	Unpaired Student’s t-test	HD versus P2	p=0.0026
	Unpaired Student’s t-test	Mo versus P1	p<0.0001
	Unpaired Student’s t-test	Mo versus P2	p=0.0011
Figure 2F	Unpaired Student’s t-test	WT versus L374P	p<0.0001
Figure 2G	Unpaired Student’s t-test	WT versus L374P	p<0.0001
Figure 2H	Mann Whitney U test	WT versus L374P	P=0.0295
Figure 3A (CD3/CD28 –FK506)	Unpaired Student’s t-test	HD versus P1	p=0.0015
Figure 3A (CD3/CD28 –FK506)	Unpaired Student’s t-test	HD versus P2	p=0.0022
Figure 3A (CD3/CD28 –FK506)	Unpaired Student’s t-test	Mother versus P1	p=0.0493
Figure 3B (CD3/CD28 –FK506)	Unpaired Student’s t-test	HD versus P1	p=0.0001
Figure 3B (CD3/CD28 –FK506)	Unpaired Student’s t-test	HD versus P2	p=0.0169
Figure 3B (CD3/CD28 –FK506)	Unpaired Student’s t-test	Mother versus P1	p=0.0111
Figure 4B (CD4+)	Unpaired Student’s t-test	HD1 versus HD1+GSK	p=0.0004
Figure 4B (CD8+)	Unpaired Student’s t-test	HD1 versus HD1+GSK	p=0.0474
Figure 4C (CD4+)	one-way ANOVA.	HD1 versus P1	p=0.0125
Figure 4C (CD4+)	one-way ANOVA.	HD1 versus HD1+GSK	p=0.0073
Figure 4C (CD4+)	one-way ANOVA.	Mother versus P1	p=0.0301
Figure 4C (CD8+)	one-way ANOVA.	HD1 versus P1	p<0.0001
Figure 4C (CD8+)	one-way ANOVA.	HD1 versus HD1+GSK	p<0.0001
Figure 4C (CD8+)	one-way ANOVA.	HD2 versus P1	p<0.0001
Figure 4C (CD8+)	one-way ANOVA.	HD2 versus HD2+GSK	p<0.0001
Figure 4D (SOCE)	Unpaired Student’s t-test	HD versus HD+GSK	p<0.0001
Figure 5A (day 7)	Unpaired Student’s t-test	WT versus Stim1VavCre	p=0.0002
Figure 5A (day 7)	Unpaired Student’s t-test	WT versus Stim1Stim2VavCre	p<0.0001
Figure 5B	Mann Whitney U test	WT versus Stim1VavCre	p<0.0001
Figure 5B	Mann Whitney U test	WT versus Stim1Stim2VavCre	p<0.0001
Figure 5E	Unpaired Student’s t-test	WT versus Stim1VavCre	P=0.0001
Figure 5E	Unpaired Student’s t-test	WT versus Stim1Stim2VavCre	p<0.0001
Figure 5G (IL-17A)	Unpaired Student’s t-test	WT versus Stim1VavCre	p=0.0002
Figure 5G (IL-17A)	Unpaired Student’s t-test	WT versus Stim1Stim2VavCre	p<0.0001
Figure 5G (IFN-γ)	Unpaired Student’s t-test	WT versus Stim1VavCre	p=0.0017
Figure 5G (IFN-γ)	Unpaired Student’s t-test	WT versus Stim1Stim2VavCre	p=0.0012
Figure 5G (IL-17F)	Unpaired Student’s t-test	WT versus Stim1VavCre	n.s.
Figure 5G (IL-17F)	Unpaired Student’s t-test	WT versus Stim1Stim2VavCre	n.s.
Figure 5G (TNFα)	Unpaired Student’s t-test	WT versus Stim1VavCre	p<0.0001
Figure 5G (TNFα)	Unpaired Student’s t-test	WT versus Stim1Stim2VavCre	p<0.0001
Figure 5G (GM-CSF)	Unpaired Student’s t-test	WT versus Stim1VavCre	p=0.0002
Figure 5G (GM-CSF)	Unpaired Student’s t-test	WT versus Stim1Stim2VavCre	p<0.0001
Figure 5H	Unpaired Student’s t-test	WT versus Stim1Mx1Cre	p=0.0903
Figure 5H	Unpaired Student’s t-test	WT versus Stim1Stim2Mx1Cre	p<0.0001
Figure 5I	Unpaired Student’s t-test	WT versus Stim1Mx1Cre	p=0.0936

Figure 5I	Unpaired Student's t-test	WT versus Stim1Stim2Mx1Cre	p=0.0592
Figure 6A (day 7)	Unpaired Student's t-test	WT versus Stim1Cd4Cre	n.s.
Figure 6B	Mann Whitney U test	WT versus Stim1Cd4Cre	n.s.
Figure 6E	Log-rank (Mantel-Cox) test	WT versus Stim1Cd4Cre	p=0.0392
Figure 6F (day 5,6)	Unpaired Student's t-test	WT versus Stim1Cd4Cre	p=0.0222
Figure 6H (Liver)	Mann Whitney U test	WT (PBS) versus Stim1Cd4Cre (Cand.)	p=0.0012
Figure 6H (Liver)	Mann Whitney U test	Stim1Cd4Cre (PBS) versus Stim1Cd4Cre (Cand.)	p=0.0012
Figure 6H (Kidney)	Mann Whitney U test	WT (PBS) versus Stim1Cd4Cre (Cand.)	p=0.0249
Figure 6H (Kidney)	Mann Whitney U test	Stim1Cd4Cre (PBS) versus Stim1Cd4Cre (Cand.)	p=0.0249
Figure 6H (Lung)	Mann Whitney U test	WT (PBS) versus Stim1Cd4Cre (Cand.)	p=0.0047
Figure 6H (Lung)	Mann Whitney U test	Stim1Cd4Cre (PBS) versus Stim1Cd4Cre (Cand.)	p=0.0047
Figure 6J (IL-2)	Unpaired Student's t-test	WT versus Stim1Cd4Cre	p=0.0341
Figure 6J (TNFα)	Unpaired Student's t-test	WT versus Stim1Cd4Cre	p=0.0268
Figure 6J (IFNγ)	Unpaired Student's t-test	WT versus Stim1Cd4Cre	p=0.0130
Figure 6J (IL-17A)	Unpaired Student's t-test	WT versus Stim1Cd4Cre	p=0.2840
Figure 6J (GM-CSF)	Unpaired Student's t-test	WT versus Stim1Cd4Cre	p=0.0282
Figure 6K	Unpaired Student's t-test	WT versus Stim1Cd4Cre	p=0.0500
Figure 7C (*Il17a*)	Unpaired Student's t-test	WT versus Stim1Cd4Cre	p<0.0001
Figure 7C (*Il17f*)	Unpaired Student's t-test	WT versus Stim1Cd4Cre	p=0.0001
Figure 7C (*Il22*)	Unpaired Student's t-test	WT versus Stim1Cd4Cre	n.s.
Figure 7C (*Il21*)	Unpaired Student's t-test	WT versus Stim1Cd4Cre	p=0.0156
Figure 7C (*Csf2*)	Unpaired Student's t-test	WT versus Stim1Cd4Cre	p=0.0243
Figure 7C (*Il1r1*)	Unpaired Student's t-test	WT versus Stim1Cd4Cre	p=0.0003
Figure 7C (*Il23r*)	Unpaired Student's t-test	WT versus Stim1Cd4Cre	p=0.0028
Figure 8B	Unpaired Student's t-test	WT versus Stim1Cd4Cre	p=0.0135
Figure 8C (Glycolysis)	Unpaired Student's t-test	WT versus Stim1Cd4Cre	p=0.0157
Figure 8C (Glycolytic capacity)	Unpaired Student's t-test	WT versus Stim1Cd4Cre	p=0.0052
Figure 8C (Glycolytic reserve)	Unpaired Student's t-test	WT versus Stim1Cd4Cre	p=0.0068
Figure 8D (Basal respiration)	Unpaired Student's t-test	WT versus Stim1Cd4Cre	p=0.0006
Figure 8D (ATP production)	Unpaired Student's t-test	WT versus Stim1Cd4Cre	p=0.0002
Figure 8D (Maximal respiration)	Unpaired Student's t-test	WT versus Stim1Cd4Cre	p=0.0005
Figure 8D (Coupling efficiency)	Unpaired Student's t-test	WT versus Stim1Cd4Cre	p=0.0005
Figure EV1 Panel C (left)	Unpaired Student's t-test	WT Unst vs WT TG	p=0.0016
Figure EV1 Panel C (left)	Unpaired Student's t-test	ΔK Unst vs ΔK TG	p=0.0007
Figure EV1 Panel C (left)	Unpaired Student's t-test	WT Unst vs L374P Unst	p=0.0316
Figure EV1 Panel C (left)	Unpaired Student's t-test	WT Unst vs L374P-ΔK Unst	p=0.0028
Figure EV1 Panel C (left)	Unpaired Student's t-test	ΔK Unst vs L374P-ΔK Unst	p=0.0436
Figure EV1 Panel C (right)	Unpaired Student's t-test	WT Unst vs WT TG	p=0.0057
Figure EV1 Panel C (right)	Unpaired Student's t-test	ΔK Unst vs ΔK TG	p=0.0405
Figure EV1 Panel C (right)	Unpaired Student's t-test	WT Unst vs L374P Unst	p=0.0044
Figure EV1 Panel C (right)	Unpaired Student's t-test	WT Unst vs L374P-ΔK Unst	p=0.0149
Figure EV1 Panel C (right)	Unpaired Student's t-test	ΔK Unst vs L374P-ΔK Unst	p=0.0378
Figure EV1E	Paired t-test	WT Unst versus WT TG	p=0.0236
Figure EV1E	Paired t-test	WT Unst versus L374P Unst	p=0.0036
Figure EV1E	Paired t-test	L374P Unst versus L374P TG	p=0.0067
Figure EV1E	Paired t-test	WT TG versus L374P TG	p=0.0131
Figure EV2A (IL-17A)	Unpaired Student's t-test	WT versus Stim1Cd4Cre	p<0.0001

Non-pathogenic			
Figure EV2A (IL-17A) Pathogenic	Unpaired Student's t-test	WT versus Stim1Cd4Cre	p=0.0394
Figure EV3 A (Il17a)	Unpaired Student's t-test	WT versus Stim1Cd4Cre	n.s.
Figure EV3 A (Il17f)	Unpaired Student's t-test	WT versus Stim1Cd4Cre	n.s.
Figure EV3 A (Il22)	Unpaired Student's t-test	WT versus Stim1Cd4Cre	n.s.
Figure EV3 A (Il21)	Unpaired Student's t-test	WT versus Stim1Cd4Cre	n.s.
Figure EV3 A (Csf2)	Unpaired Student's t-test	WT versus Stim1Cd4Cre	n.s.
Figure EV3 A (Il1r1)	Unpaired Student's t-test	WT versus Stim1Cd4Cre	p=0.0177
Figure EV3 A (Il23r)	Unpaired Student's t-test	WT versus Stim1Cd4Cre	n.s.
Figure EV3 B (Il10) Non-pathogenic	Unpaired Student's t-test	WT versus Stim1Cd4Cre	n.s.
Figure EV3 B (Il10) Pathogenic	Unpaired Student's t-test	WT versus Stim1Cd4Cre	p=0.0011
Figure EV3 B (Rorc) Non-pathogenic	Unpaired Student's t-test	WT versus Stim1Cd4Cre	n.s.
Figure EV3 B (Rorc) Pathogenic	Unpaired Student's t-test	WT versus Stim1Cd4Cre	p=0.0022
Figure EV3 B (Ifng) Non-pathogenic	Unpaired Student's t-test	WT versus Stim1Cd4Cre	n.s.
Figure EV3 B (Ifng) Pathogenic	Unpaired Student's t-test	WT versus Stim1Cd4Cre	n.s.
Figure EV3 B (Il4) Non-pathogenic	Unpaired Student's t-test	WT versus Stim1Cd4Cre	n.s.
Figure EV3 B (Il4) Pathogenic	Unpaired Student's t-test	WT versus Stim1Cd4Cre	p=0.0062
Figure EV3 B (Il13) Non-pathogenic	Unpaired Student's t-test	WT versus Stim1Cd4Cre	n.s.
Figure EV3 B (Il13) Pathogenic	Unpaired Student's t-test	WT versus Stim1Cd4Cre	p=0.0344
Figure EV3 B (Il9) Non-pathogenic	Unpaired Student's t-test	WT versus Stim1Cd4Cre	p=0.0223
Figure EV3 B (Il9) Pathogenic	Unpaired Student's t-test	WT versus Stim1Cd4Cre	p=0.0373
Figure EV3 C (Slc2a3)	Unpaired Student's t-test	WT versus Stim1Cd4Cre	n.s.
Figure EV3 C (Slc2a1)	Unpaired Student's t-test	WT versus Stim1Cd4Cre	n.s.
Figure EV3 C (Hk1)	Unpaired Student's t-test	WT versus Stim1Cd4Cre	n.s.
Figure EV3 C (Hk2)	Unpaired Student's t-test	WT versus Stim1Cd4Cre	n.s.
Figure EV3 C (Aldoa)	Unpaired Student's t-test	WT versus Stim1Cd4Cre	n.s.
Figure EV3 C (Gpi1)	Unpaired Student's t-test	WT versus Stim1Cd4Cre	n.s.
Figure EV3 C (Ldha)	Unpaired Student's t-test	WT versus Stim1Cd4Cre	n.s.
Figure EV3 C (Slc16a1)	Unpaired Student's t-test	WT versus Stim1Cd4Cre	n.s.
Figure EV3 C (Gapdh)	Unpaired Student's t-test	WT versus Stim1Cd4Cre	n.s.
Figure EV3 C (Pgk1)	Unpaired Student's t-test	WT versus Stim1Cd4Cre	n.s.
Figure EV3 C (Pfkfb1)	Unpaired Student's t-test	WT versus Stim1Cd4Cre	n.s.
Figure EV3 C (Pfkfb4)	Unpaired Student's t-test	WT versus Stim1Cd4Cre	n.s.
Figure EV3 C (Pfkl)	Unpaired Student's t-test	WT versus Stim1Cd4Cre	n.s.
Figure EV3 C (Pfkm)	Unpaired Student's t-test	WT versus Stim1Cd4Cre	n.s.
Figure EV3 C (Tpi1)	Unpaired Student's t-test	WT versus Stim1Cd4Cre	n.s.
Figure EV3 C (Pfkp)	Unpaired Student's t-test	WT versus Stim1Cd4Cre	n.s.
Figure EV3 C (Pkm)	Unpaired Student's t-test	WT versus Stim1Cd4Cre	n.s.

Appendix Table S3: Exact p-values for data shown in Figures 1-8 and EV1-4. n.s. = not significant
