# Supplementary material for: STIM1‐mediated calcium influx controls antifungal immunity and the metabolic function of non‐pathogenic Th17 cells
Source: EMBO Mol Med. 2020 Jul 1;12(8):e11592. doi: 10.15252/emmm.201911592 (PMC7411566; doi:10.15252/emmm.201911592)
Supplement: Supplementary file 2 — Expanded View Figures PDF [file EMMM-12-e11592-s002.pdf]

Expanded View Figures

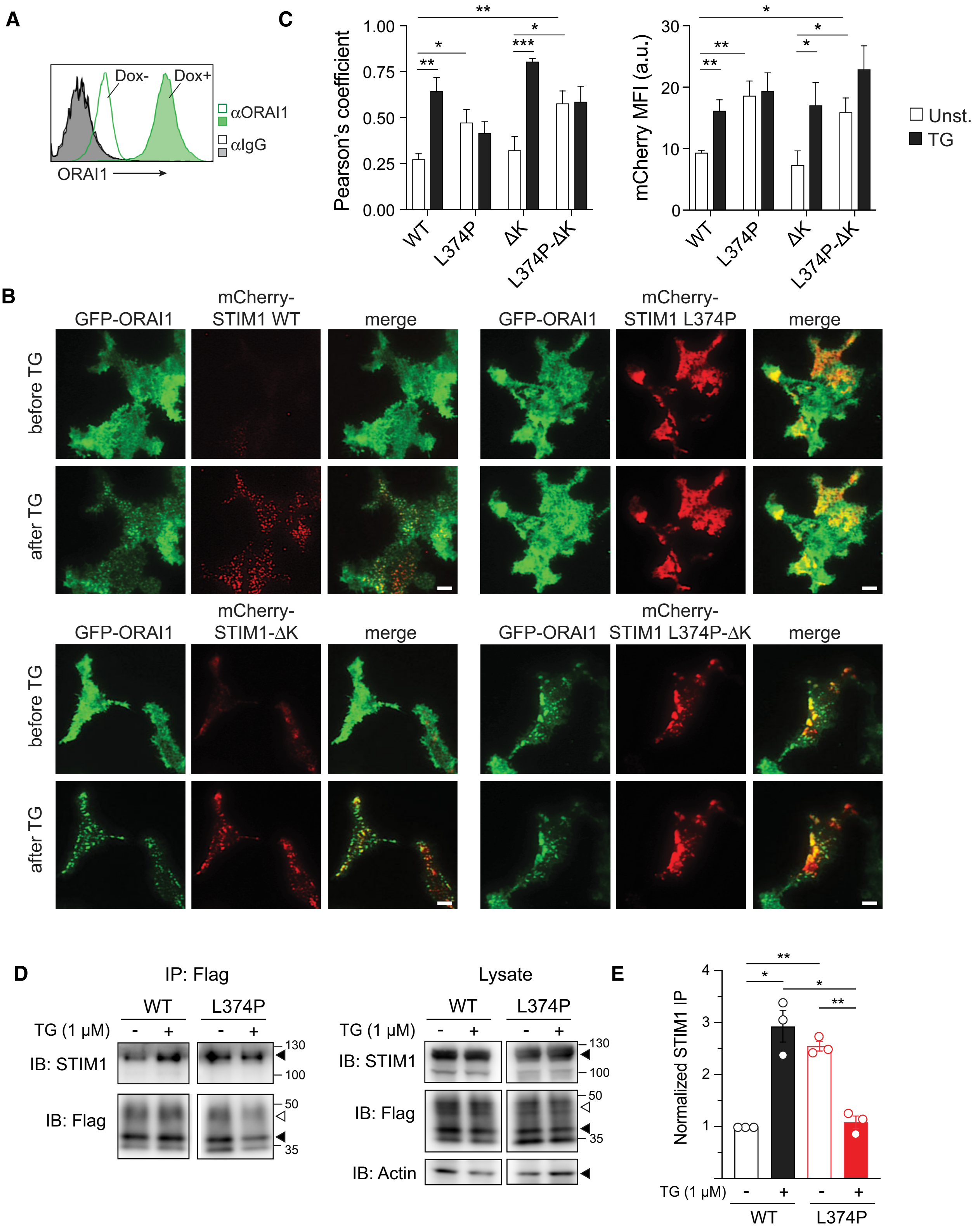

Figure EV1.

© 2020 The Authors

Figure EV1. Plasma membrane localization of STIM1 p.L374P mutant is independent STIM1 polybasic domain and ORAI1 expression.

A Representative flow cytometry plots of ORAI1 expression on HEK293 cells after doxycycline (Dox)-induced overexpression of ORAI1 for 24 h. ORAI1 was detected using a mouse anti-human monoclonal antibody (29A2) recognizing the second extracellular loop of ORAI1. Isotype control: mouse IgG1. Open histograms (no Dox) represent controls.

B TIRFM images of HEK293 cells overexpressing GFP-ORAI1 and mCherry-STIM1 (either WT, L374P, ΔK, or L374P-ΔK). Cells were analyzed before or after stimulation with 1 μM TG in Ca^{2+}-free Ringer's buffer for 10 min. Representative images from one of 3–4 repeat experiments. Scale bars, 10 μm.

C (left panel) Co-localization of mCherry-STIM1 (WT or mutants) and GFP-ORAI1 before and 10 min after treatment with TG. Co-localization was measured using Pearson's coefficient for GFP and mCherry. (right panel) Mean fluorescence intensity (MFI) of WT or mutant mCherry-STIM1 in the TIRFM evanescent field before and after TG stimulation as in (B). Data are the mean ± SEM from 9 to 14 cells from 3 to 4 independent experiments per condition.

D Co-immunoprecipitation of mCherry-STIM1 (WT or L374P) and Flag-ORAI1 coexpressed in HEK293 cells in resting condition or after TG stimulation (1 μM). STIM1 and ORAI1 were detected by IB using anti-STIM1 (3917) and anti-Flag (M2) antibodies. Shown is one representative experiment of three. Filled triangles indicate bands for mCherry-STIM1, Flag-ORAI1 and actin; open triangles indicate bands of glycosylated Flag-ORAI1.

E The immunoprecipitated (IP) mCherry-STIM1 signal was normalized to total protein content (actin), total mCherry-STIM1 expression, and IP efficiency (Flag-ORAI1). Shown is the mean ± SEM of three independent experiments.

Data information: Statistical analyses in (C) and (E) by unpaired Student's *t*-test. $*P < 0.05$, $**P < 0.01$, $***P < 0.001$.

Source data are available online for this figure.

Figure EV2. RNA sequencing of non-pathogenic and pathogenic Th17 cells.

A IL-17A and IFN-γ expression of $CD4^+$ T cells isolated from WT and *Stim1*$^{fl/fl}$*Cd4Cre* mice, differentiated into non-pathogenic (np) and pathogenic (p) Th17 cells for 2 days and then analyzed by RNA sequencing. Data are the mean ± SEM from 3 mice per genotype.

B Principal component analysis (PCA) of $CD4^+$ T cells from three WT and three *Stim1*$^{fl/fl}$*Cd4Cre* mice differentiated into non-pathogenic npTh17 and pTh17 cells *in vitro*. Each dot represents one biological replicate (donor mouse).

C Volcano plots of differentially expressed genes (DEGs) in pathogenic Th17 cells. Highlighted in red (upregulated in *Stim1*$^{fl/fl}$*Cd4Cre*) and blue (downregulated in *Stim1*$^{fl/fl}$*Cd4Cre*) are DEGs that belong to a gene expression signature of npTh17 cells (left plot) and pTh17 cells (right plot) defined previously (Lee *et al*, 2012; Gaublomme *et al*, 2015). The corresponding DEG analysis of npTh17 cells is shown in Fig 7B and C.

Data information: Statistical analysis in (A) by unpaired Student's *t*-test with the following significance levels: $*P < 0.05$, $***P < 0.001$; in (C) by Wald *t*-test corrected by Benjamini–Hochberg method.

© 2020 The Authors

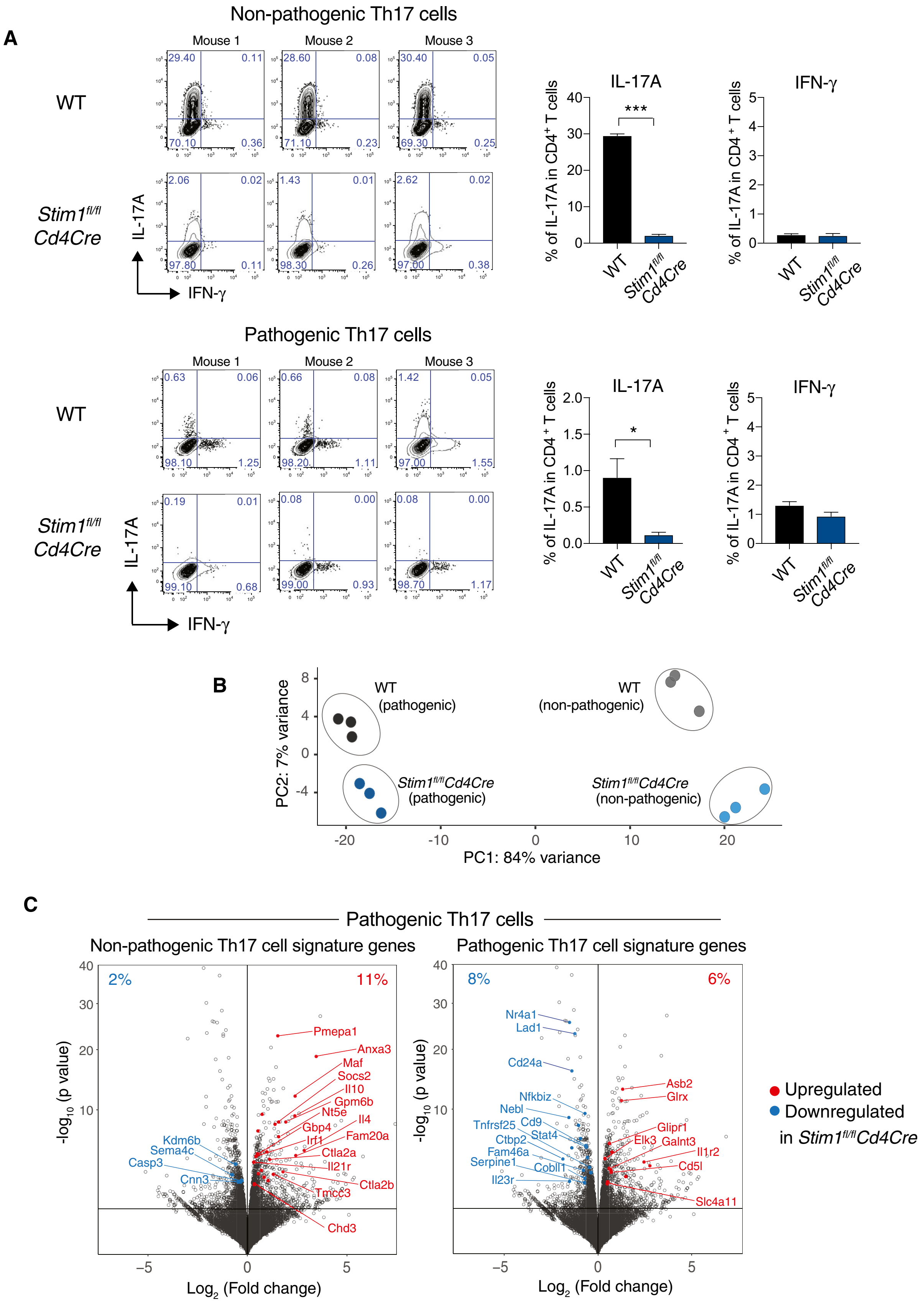

Figure EV2.

© 2020 The Authors

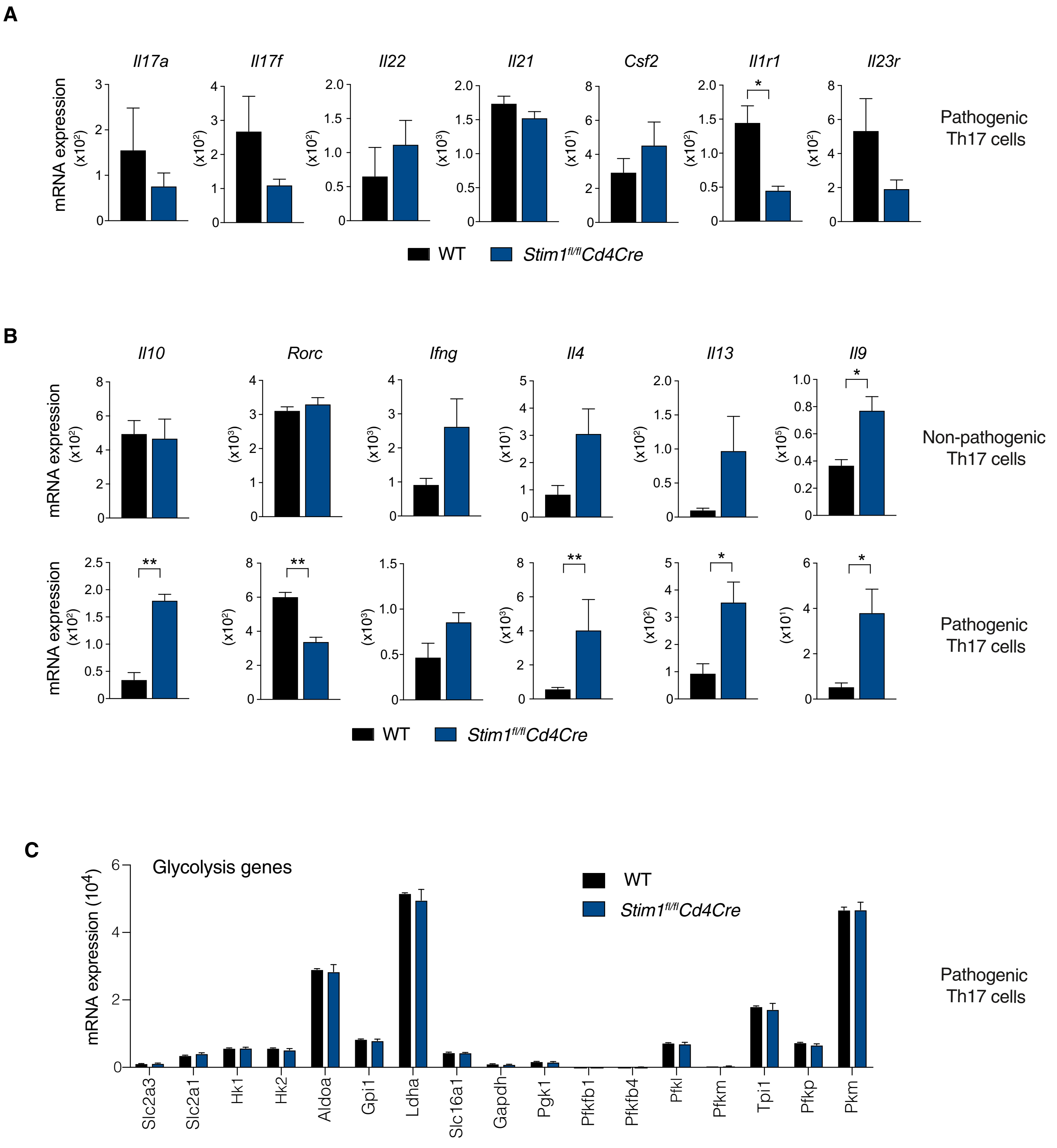

Figure EV3. Expression of cytokines, cytokine receptors, and glycolysis-associated genes in STIM1-deficient Th17 cells.

A, B Analysis of mRNA expression of the indicated cytokines, cytokine receptors, and transcription factors in $CD4^+$ T cells from WT and *Stim1*$^{fl/fl}$*Cd4Cre* mice that were differentiated into pathogenic (A) or pathogenic and non-pathogenic (B) Th17 cells *in vitro* for 2 days and then analyzed by RNA sequencing.

C Analysis of mRNA expression of glycolytic enzymes in $CD4^+$ T cells from WT and *Stim1*$^{fl/fl}$*Cd4Cre* mice that were differentiated into pathogenic Th17 cells *in vitro* for 2 days and then analyzed by RNA sequencing.

Data information: Data in (A-C) are the mean ± SEM from 3 mice per genotype. Statistical analysis by unpaired Student's *t*-test with the following significance levels: *$P < 0.05$, **$P < 0.01$.

© 2020 The Authors

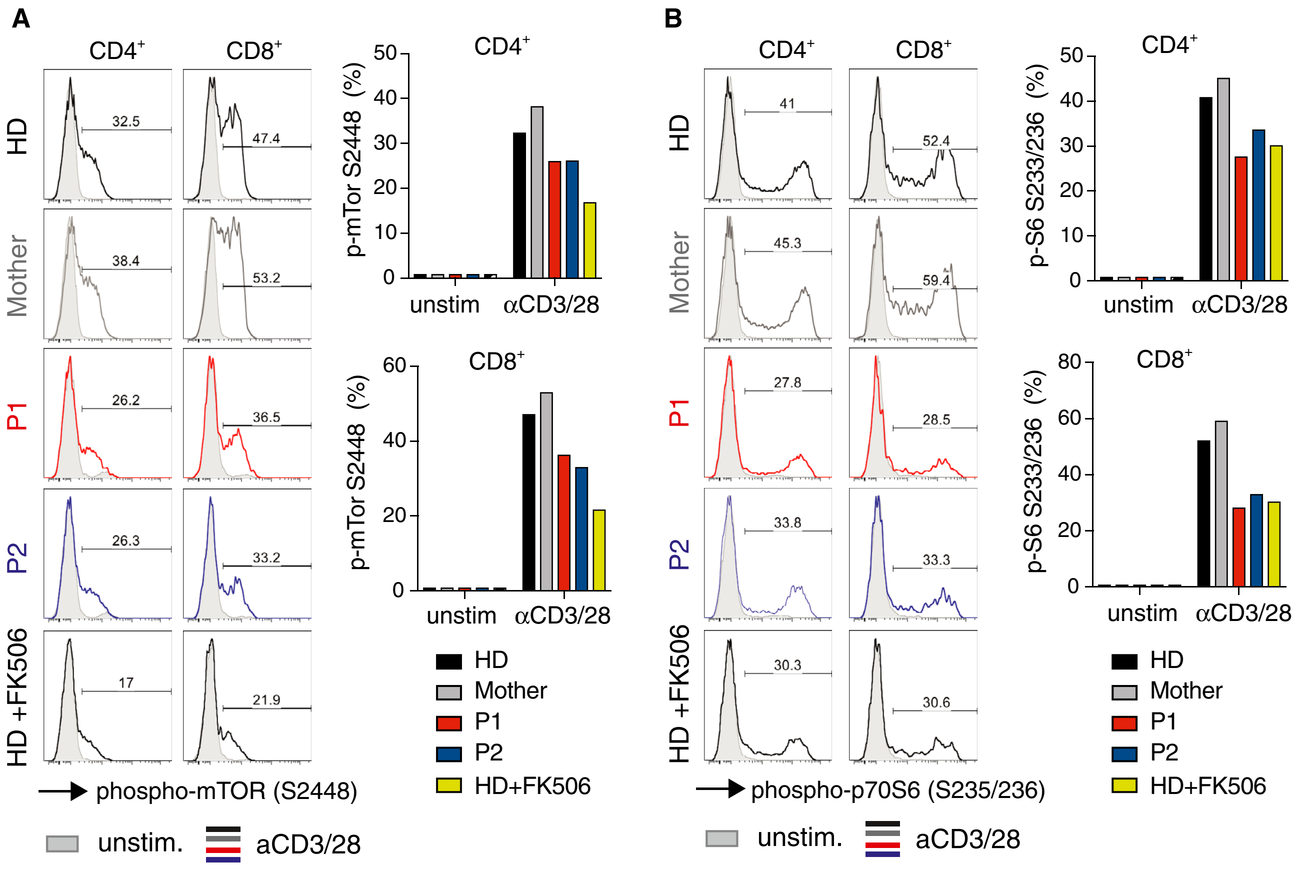

Figure EV4. Reduced mTOR and S6 phosphorylation in T cells of STIM1-deficient patients isolated *ex vivo*.

A, B Analysis of mTOR and p70S6 phosphorylation in $CD4^+$ and $CD8^+$ T cells freshly isolated from PBMC of P1 (red), P2 (blue), their mother (gray), and a HD (black) and stimulated with anti-CD3/CD28 for 24 h or left unstimulated. HD T cells were stimulated in the presence or absence of 1 μM FK506. Histograms represent intracellular staining of cells with anti-phospho-mTOR (Ser2448) (A) and anti-phospho-p70S6 (Ser235/236) (B) antibodies. Filled gray histograms represent unstimulated cells. Numbers above gates in histograms and in bar graphs indicate the frequencies of $CD4^+$ and $CD8^+$ T cells positive for phospho-mTOR and phospho-p70S6 staining.

© 2020 The Authors
