# Supplementary material for: STIM1‐mediated calcium influx controls antifungal immunity and the metabolic function of non‐pathogenic Th17 cells
Source: EMBO Mol Med. 2020 Jul 1;12(8):e11592. doi: 10.15252/emmm.201911592 (PMC7411566; doi:10.15252/emmm.201911592)
Supplement: Supplementary file 3 — Table EV1 [file EMMM-12-e11592-s003.doc]

| **Patient** | **P1** (A-II-1) | **P2** (A-II-2) |
| --- | --- | --- |
| **Inheritance** | AR | AR |
| **Type of mutation** | Missense c.1121T>C | Missense c.1121T>C |
| **STIM1 mutation** | p.L374P | p.L374P |
| **STIM1 expression** | mRNA and protein normal | mRNA and protein normal |
| **SOCE** | strongly reduced | strongly reduced |
| **SOCE defect** (tested in) | PBMC, T cells *in vitro* | PBMC, T cells *in vitro* |
| **Infections** | Pneumonias caused by Mycobacterium heckeshornense; Varizella zoster virus (VZV) infections | Recurrent typical pneumonias; recurrent bacterial otitis media; onychomycosis and skin infections with *C. albicans* species |
| **Autoimmunity** | n.r. | Crohn’s disease |
| **Immune cell subsets** | normal | normal |
| **Lymphocyte function** | T cell proliferation ↓  T cell cytokine production ↓ | T cell proliferation ↓  T cell cytokine production ↓ |
| **Antibodies** | Borderline low serum IgG levels;  strongly reduced numbers of class-switched (IgG^+^) CD27^+^ memory B cells | Normal Ig subclasses; no seroconversion following rubella vaccination; strongly reduced numbers of class-switched (IgG^+^) CD27^+^ memory B cells |
| **Myopathy** | Congenital muscular hypotonia with type 2 muscle fiber atrophy, partial aniridia with mydriasis | Congenital muscular hypotonia with type 2 muscle fiber atrophy, partial aniridia with mydriasis |
| **Anhidrotic ectodermal dysplasia (EDA)** | Anhidrosis, dental enamel hypoplasia,  mild hypotrichosis | Anhidrosis, dental enamel hypoplasia,  mild hypotrichosis |
| **Other complications** | Hypercalciuria, hypercalcaemia;  psoriasiform eczematoid skin reaction; EBV related lymphoproliferative disorder ay 24 y/o (most consistent with a diffuse large B-cell lymphoma) | Hepatosplenomegaly, cervical lymphadenopathy,  malabsorption, steatorrhea, hypercholesterinemia, hypertriglyceridemia, hypercalcaemia, hypercalciuria, microcytic hypochromic anemia |
| **Outcome** | Death at 25 y/o from pneumonia | Alive at 31 y/o without HSCT; gave birth to healthy daughter at 29 y/o |

**Table EV1:** Summary of clinical and laboratory findings in P1 and P2. Abbreviations: AR, autosomal recessive; HSCT, Hematopoietic stem cell transplantation; Ig, Immunoglobulin; n.r., not reported; y/o, years old.
