# Supplementary figures and images for: STIM1‐mediated calcium influx controls antifungal immunity and the metabolic function of non‐pathogenic Th17 cells
Source: EMBO Mol Med. 2020 Jul 1;12(8):e11592. doi: 10.15252/emmm.201911592 (PMC7411566; doi:10.15252/emmm.201911592)

Figure EV1

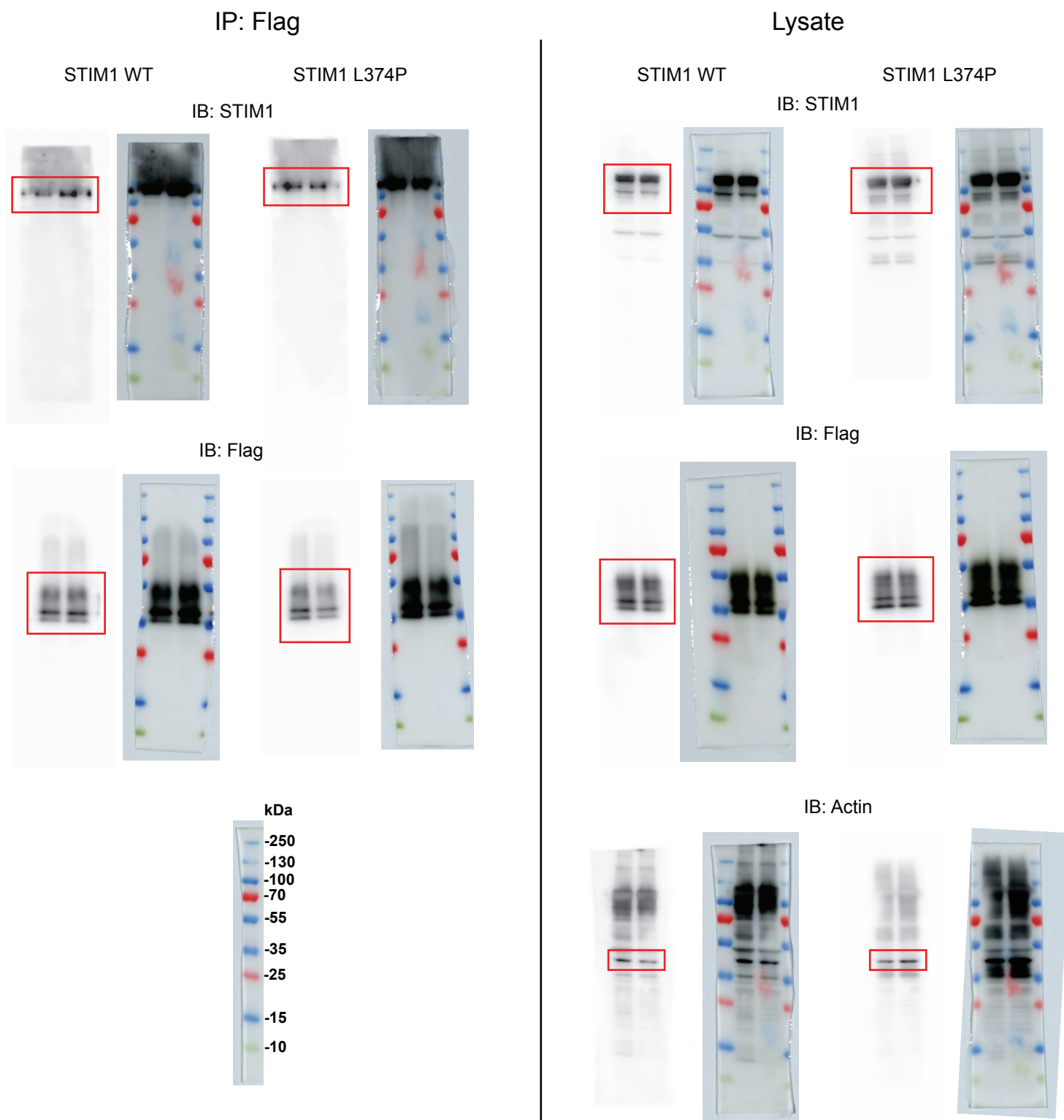

Supplement: Supplementary file 4 — Source Data for Expanded View [file EMMM-12-e11592-s005.zip › emmm201911592-sup-0005-SDataEV/emmm201911592-sup-0005-SDataFigEV1.pdf]

Figure 1

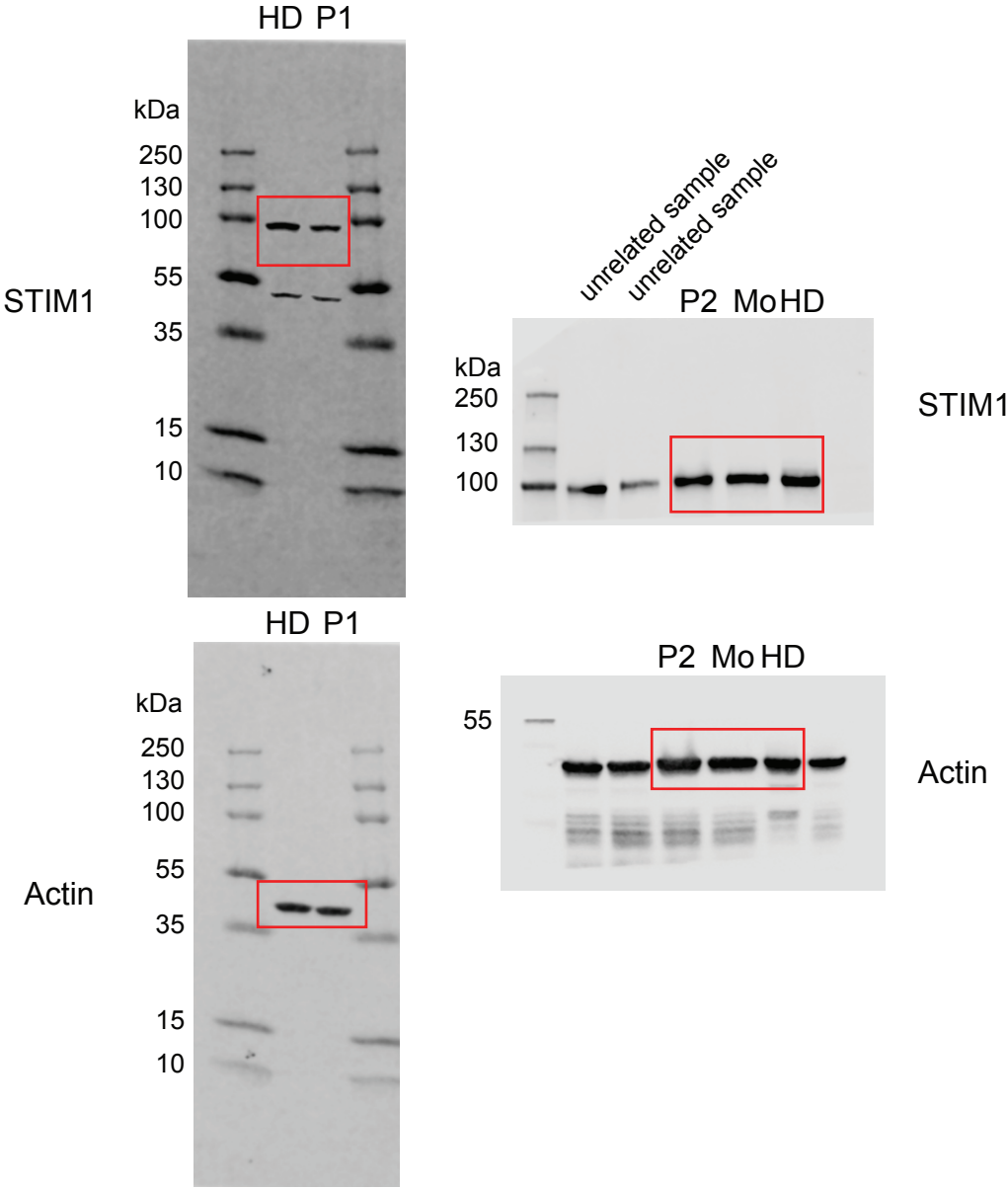

Supplement: Supplementary file 6 — Source Data for Figure 1 [file EMMM-12-e11592-s004.pdf]
